# Maturation of Lymphocyte Immunophenotypes and Memory T Helper Cell Differentiation During Development in Mice

**DOI:** 10.1155/2000/56106

**Published:** 2000

**Authors:** Omar R. Fagoaga, Steven. M. Yellon, Sandra. L. Nehlsen-Cannarella

**Affiliations:** 1 lmmunology Center Department of Pathology Loma Linda University School of Medicine and Medical Center 11234 Anderson St. Room 2578 Loma Linda CA 92354-2870 USA; 2 Immunology Center Department of Physiology Loma Linda University School of Medicine and Medical Center Loma Linda CA 92354-2870 USA

**Keywords:** development, immunophenotype, mouse, neonate, thymic-exportation

## Abstract

The goal of this study was to systematically investigate the ontogeny of lymphoid populations throughout postnatal development. In CD-1 mice, peak lymphocyte numbers occurred in blood on postnatal day 10 (dl0) including those for natural killers (NK1.1), B cells (CD19), T helper (CD3CD4), naïve T helper (CD4CD62L^pos^CD44^low^), memory T helper (CD4CD62L^neg^CD44^high^), and T cytotoxic (CD3CD8) cells. As percent of total lymphocytes, peaks were achieved by d10 for all T helper subtypes but not B cells which declined to a nadir. In spleen, lymphocyte numbers increased exponentially after d10. Proportionately, NK and T cells peaked on d10, declined by d20, and increased 2–3-fold by d45. Naive T cells constituted the majority of lymphocytes during development while memory cells gained to 2.2% (blood) and 12 % (spleen) by d20. C57BL/6 mice had similar profiles except that the B cell nadir and T cell subset peaks were at d5. Peripheralization of critical numbers of lymphocytes by d10, and importantly, development of a repertoire of memory cells by d20, may define immune response capabilities that close the period of immaturity for the neonate.


*Developmental Immunology*, 2000, Vol. 8(1), pp. 47-60
Reprints available directly from the publisher
Photocopying permitted by license only

© 2000 OPA (Overseas Publishers Association)
N.V. Published by license under the
Harwood Academic Publishers imprint,
part of The Gordon and Breach Publishing Group.
Printed in Malaysia

### Maturation of Lymphocyte Immunophenotypes and Memory T Helper Cell Differentiation During Development in Mice

OMAR R. FAGOAGA[a], STEVEN. M. YELLON[b] and SANDRA. L. NEHLSEN-CANNARELLA[ab*†]

[a]*Immunology Center, Department of Pathology and* [b]*Department of Physiology, Loma Linda University School of Medicine and Medical Center, Loma Linda, CA 92354–2870 USA*

*(Received September 07, 1999; Revised September 16, 1999)*

The goal of this study was to systematically investigate the ontogeny of lymphoid populations throughout postnatal development. In CD-1 mice, peak lymphocyte numbers occurred in blood on postnatal day 10 (d10) including those for natural killers (NK1.1), B cells (CD19), T helper (CD3CD4), naïve T helper (CD4CD62L$^{pos}$CD44$^{low}$), memory T helper (CD4CD62L$^{neg}$CD44$^{high}$), and T cytotoxic (CD3CD8) cells. As percent of total lymphocytes, peaks were achieved by d10 for all T helper subtypes but not B cells which declined to a nadir. In spleen, lymphocyte numbers increased exponentially after d10. Proportionately, NK and T cells peaked on d10, declined by d20, and increased 2–3-fold by d45. Naive T cells constituted the majority of lymphocytes during development while memory cells gained to 2.2% (blood) and 12 % (spleen) by d20. C57BL/6 mice had similar profiles except that the B cell nadir and T cell subset peaks were at d5. Peripheralization of critical numbers of lymphocytes by d10, and importantly, development of a repertoire of memory cells by d20, may define immune response capabilities that close the period of immaturity for the neonate.

*Keywords:* development, immunophenotype, mouse, neonate, thymic-exportation

### INTRODUCTION

Survival is dependent on at least two conditions, the presence of immune and accessory cells (appropriate proportions and numbers), and functional capacity. Specific lymphocyte subsets in circulation and secondary lymphoid organs define host defense capabilities and the status of immune activity (Barclay *et al.*, 1997; Bikoue *et al.*, 1997). In the human, fewer T cells are present in umbilical blood compared to that in 5-day-old neonates or adults (Pittard *et al.*, 1989; Raes *et al.*, 1993). Compared to later ages, neonates have lower numbers of T lymphocytes (Pittard *et al.*, 1989; Han *et al.*, 1995). Newborns have 10 times more naïve (CD4CD45RA+) cells than adults (Raes *et al.*, 1993; Han *et al.*, 1995). However, during

* To whom proofs should be sent: S. L. Nehlsen-Cannarella, PhD, Immunology Center, Loma Linda University Medical Center, 11234 Anderson Street, Loma Linda, CA 92354–2870

† Address correspondence and reprint requests to Dr. S. Nehlsen-Cannarella. Department of Pathology, Immunology Center, 11234 Anderson St. Room 2578, Loma Linda, CA, 92354–2870. Phone: (909) 558–8181; Fax: (909) 558–0144; e-mail: sncannarella@ahs.llumc.edu infancy (young child) CD4, memory T helpers, CD8, immunoglobulin secreting B cells, and CD5+ B cell numbers are greater than those found in adulthood (Nahmias *et al.*, 1995). The paucity of differentiated and activated lymphocyte subsets in human neonates suggests immaturity in inflammatory Th1 response capabilities in the developing immune system. In spite of these observations, immune system maturation in the human cannot yet be described in terms of immunophenotype proportions and numbers due to inadequate data collection during postnatal development.

Several studies in the mouse have focused on immunophenotype maturation but, again, neonatal ages for comparisons are limited. By d35, B cell numbers and immunophenotypes have attained adult levels some 3 weeks in advance of T cells (King *et al.*, 1991). The percentage of splenocytes (Thy 1.2, CD4, CD5, and CD8) have increased 4–10 fold from d5 to d35 (King *et al.*, 1991). Similarly in germ free and specific pathogen free mice, adult-like numbers and proportions of T helper, T cytotoxic, and B cells in spleen were achieved between d14 and d21 (Forni *et al.*, 1988). These last findings raise the possibility that development of the splenic lymphocyte repertoire is not solely driven by pathogens encountered postnatally but may also reflect an innate program of immune cell production. Further, accessory cells are required to provide appropriate signals and humoral support for optimal lymphocyte function, and these two major classes of leukocytes must be able to cooperate in providing optimal host protection.

Assessment of immune system maturity first involves the building of a catalogue of cell types populating various lymphoid compartments, and then the configuration of functional capabilities. Advent of more sensitive flow cytometric methodology and availability of extensive immunophenotyping reagents provided the means to address gaps in the current literature concerning lymphocyte differentiation during the first few postnatal weeks in the mouse. Specifically, the profile of naïve or memory T helper phenotypes, cells that are recognized as important for alloreactivity and tolerance, have not been studied during the neonatal period in any strain of mouse. Thus, the objective of the present study was to determine the ontogenic pattern (proportions and absolute numbers) of select lymphocyte subsets in blood and spleen from birth to adulthood.

### RESULTS

### Leukocytes in Blood and Spleen during Development

In CD-1 mice, a dramatic peak was found in number of leukocytes in blood on postnatal day 10 compared to a gradual increase of cells in the spleen by d20. In blood, total leukocyte concentrations peaked at 10 days of age (Figure 1, top panel; ANOVA, $p<0.05$). Cell numbers declined to a nadir by d45 (adulthood), significantly reduced compared to that at birth. In contrast, lymphocyte counts were initially elevated over those at birth by d3 and continued to increase to the peak on d10. Subsequently, cell numbers declined to a plateau by d20 that persisted into adulthood. The gain in body weight beyond day 10 was not accompanied by increased concentrations of leukocytes or lymphocytes in blood (Figure 1, bottom panel inset).

In the spleen, total leukocytes (per whole spleen) increased exponentially beginning on d5 (Figure 1, bottom panel; ANOVA, $p<0.05$). Leukocytes increased to adult-like values by d20 while lymphocyte numbers continued to increase to d45. By comparison, age-related gains in spleen weight were correlated with the rise in body weight and paralleled the profile of leukocyte and lymphocyte concentrations in spleen (Figure 1, bottom panel inset). Thus, the size of the spleen was directly related to number of resident lymphocytes. In addition, lymphocyte numbers in blood constituted 10%, 35%, and 70% of all leukocytes on d0-d3, d4-d20, and d45, respectively. By contrast, lymphocytes in spleen constituted 35%, 60% and 82% of splenocytes on d0-d5, d10-d20, and d45, respectively.

### Lymphocyte Subsets in Blood and Spleen during Development

To determine the predominant lymphocyte subset during development, specific antibodies were used to

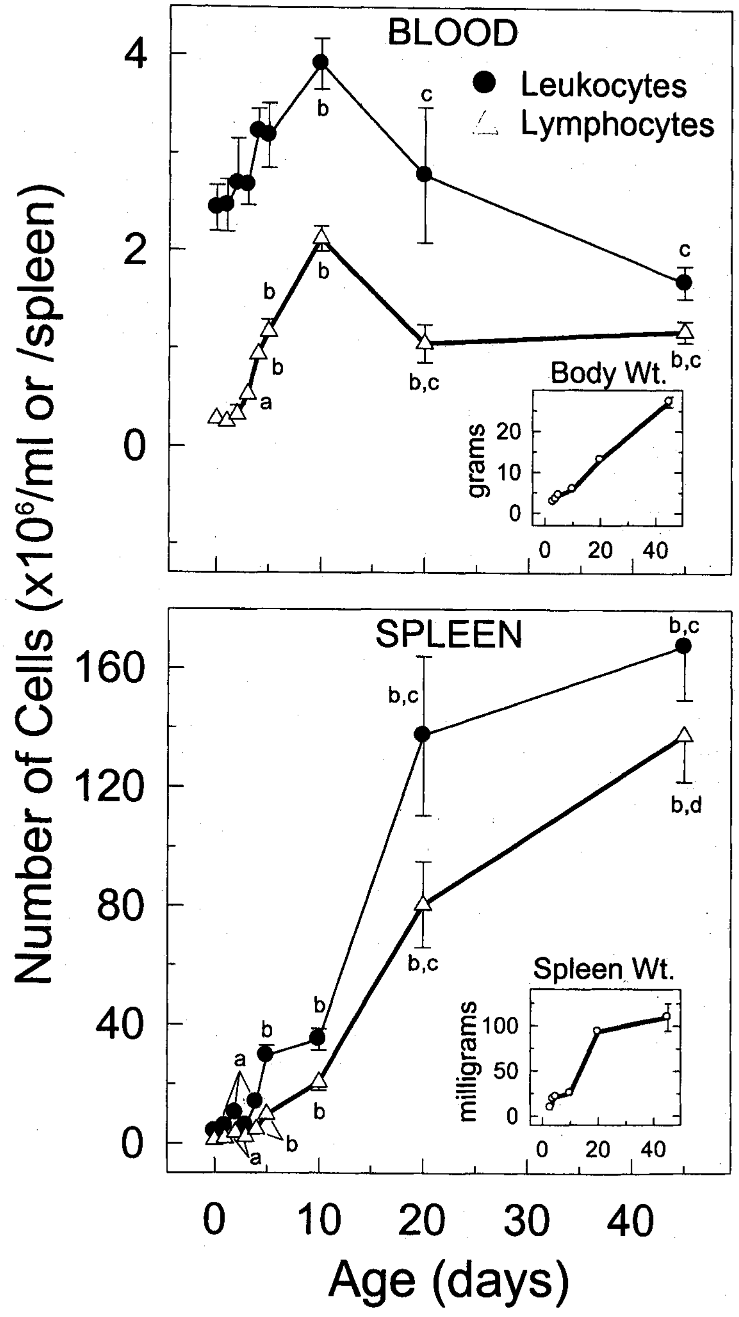

FIGURE 1 Number of leukocytes (solid circles) and lymphocytes (open triangles) in blood (top) and spleen (bottom) of CD-1 mice (male and female) at various ages during postnatal development. Data points are mean±SE of 6 samples. Samples represent pools of pups (days 0–2, 8/pool; d3, 24/pool; d4, 13/pool; d5, 10/pool; d10, 9/pool, d20, 5/pool), or individual mice (d45). Note scale difference for cell numbers of blood (per mL) compared to that of spleen (per spleen). Statistical differences ($p<0.05$) among age groups are indicated by "a" *versus* d0, "b" *versus* days 0–3, "c" *versus* d10, "d" *versus* d20. Numbers of lymphocytes in blood on d10 is different from all previous age groups. For this data set (df=8), the one-way ANOVA with Duncan's multiple range test generated an F statistic >3.72, while the Kruskal-Wallis test with Chi Square analysis was >74. See statistical analysis section in Materials and Methods for further details. Inset graphs are body and spleen weights at each postnatal age. At some ages, symbols obscure SE bars identify NK (NK1.1), B (CD20), T (CD3), T helper (CD3CD4), and T cytotoxic (CD3CD8) cells in blood and spleen. The percentage of NK1.1 cells in blood and spleen increased to a peak by d4 and d5, respectively, but this subtype represented fewer than 5% of all lymphocytes (Figure 2, left panels, ANOVA, p<0.05). Compared to the newborn, the approximate 1.5-fold increase by d4 in blood and d5 in spleen was followed by a sharp decrease to the nadir on d10 in both compartments. Thus, the proportion of NK cells in circulation and resident in spleen varied over a narrow range (1–5%) during development.

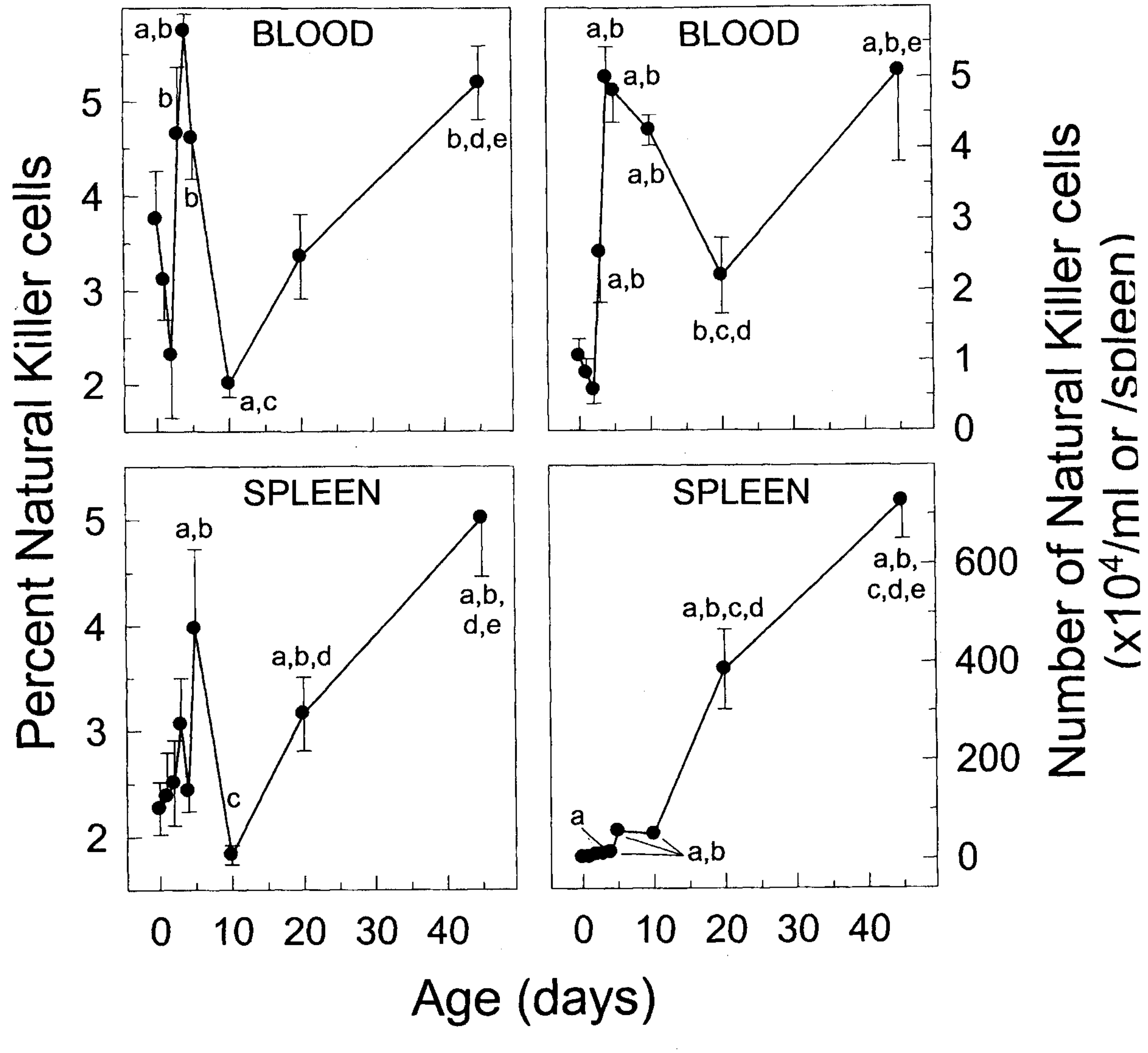

FIGURE 2 Percent (left panels) and numbers (right panels) of natural killer cells relative to total lymphocytes in blood (top panels) and spleen (bottom panels) of CD-1 mice (male and female) at various ages during postnatal development. Data are mean±SE of 6 samples. Construction of pools is described in Figure 1. Statistical differences (p <0.05) among age groups are indicated by "a" versus d0, "b" versus d2, "c" versus d5, "d" versus d10, and "e" versus d20. For this data set (df=8), one-way ANOVA with Duncan's multiple range test generated an F statistic 6.8, while Kruskal-Wallis test with Chi Square analysis was >26.6. At some ages, symbols obscure SE bars

Numbers of NK cells in blood paralleled the profile of NK percentages throughout development. In blood, NK cell numbers on d4 and d45 were significantly elevated compared to all other days (Figure 2, top right panel; ANOVA, $p<0.05$). In spleen, these cells gradually increased during the neonatal period, then rose exponentially after d10 (Figure 2, bottom right panel). Ultimately in adulthood, the magnitude of splenic NK cell numbers was some 280-fold greater than at birth.

During this same period, the B cell (CD19) population declined relative to total lymphocytes in blood and spleen (ANOVA, $p<0.05$; Figure 3, left panels). Relative proportions of CD19+ cells were high in the newborn (Day 0: blood = 90 %, spleen = 97 %). Subsequently, the proportions decreased over the first 10 days (blood = 35 %, spleen 78 %). Thereafter in blood, the B cells had increased to a plateau on d20 that persisted through d45; in the spleen, the proportions gradually but significantly decreased by d10, but had risen again to 88% by d20. Beyond that, proportions decreased to about 60% by d45.

Absolute B cell numbers increased with age in both blood and spleen (ANOVA, $p<0.05$; Figure 3, right panels). In blood, B cell numbers presented the first major increase from birth by d4, then continued to increase to a peak on d10. By d20, cell numbers had decreased to a plateau that persisted through d45. In spleen, the first significant rise from birth occurred on d2, followed by an exponential increase by d20 (90-fold over newborn values), when the numbers plateau into adulthood. Adult-like concentrations of B cells in blood and spleen were achieved by d20, the same numbers observed on d45.

The T lymphocytes (CD3) were studied with respect to their two major subtypes, T helper (CD3CD4) and T cytotoxic (CD3CD8) cells. In blood, the percentage of total T and both subtypes increased to a peak on d10 (Figure 4, left top panel). The initial rise in T cells occurred on day 3, predominantly due to expansion of the T helper cell subset (ANOVA, $p<0.05$); T cytotoxic cells had risen on d1 (ANOVA, $p<0.05$). In the spleen, as well, all T cells peaked on d10; the initial rise in total T and T cytotoxic cells occurred on d3 and d2, respectively, while T helper cells had increased on d1 (Figure 4, left bottom panel). Then the proportion of T cells in blood declined to a nadir on d20, a level not significantly different from that found at d45.

As with percentages, absolute numbers of T and T cell subsets in circulation reached peak numbers at d10 (ANOVA, $p<0.05$; Figure 4, right top panel). Compared to newborns, the initial rise in blood occurred on d3 for all T cells and represented a >50-fold increase. Their numbers declined by d20 to those found in adults. The developmental profiles for T cell proportions and numbers in blood are similar, both peak at 10 days of age. In contrast, the profile of T cell percentages in the spleen peak at d10, while absolute numbers of T cells increase exponentially from birth.

Within the spleen, exponential increases in absolute numbers of T and T cell subsets continued throughout postnatal development (Figure 4; right bottom panel). The initial rise in T helper and T cytotoxic cell numbers was evident on d1 (ANOVA, $p<0.05$). Thereafter, T cell numbers increased exponentially with age. Although the greatest rise occurred in T helper cell numbers, the sum of T helper and T cytotoxic cells at each age (except d1 and d2) was always less than CD3 T cells, suggesting the presence of γδ T cells in both compartments (Bluestone *et al.*, 1991; Tough and Sprent, 1998; Whitherden *et al.*, 1994).

### Differentiation of T Helper Lymphocytes in Blood and Spleen during Development

The proportion of T helper cells of the naïve ($CD62L^{pos}CD44^{low}$) and memory ($CD62L^{neg}CD44^{high}$) subtypes were inversely correlated in blood and spleen up to 20 days of age (Figure 5, left top panel). In blood, the steepest rise in cells that express the naive phenotype occurred on d4, a 3.6-fold increase compared to that on d3. Naïve T helper cells increased to d10, reaching a level apparently sustained through d45 (ANOVA, $p<0.05$). In contrast, memory cells declined to a nadir on d10, then increased by d45 to proportions exceeding those in the neonate. Even so, memory cells constituted less than 3% of lymphocytes in circulation. In spleen,

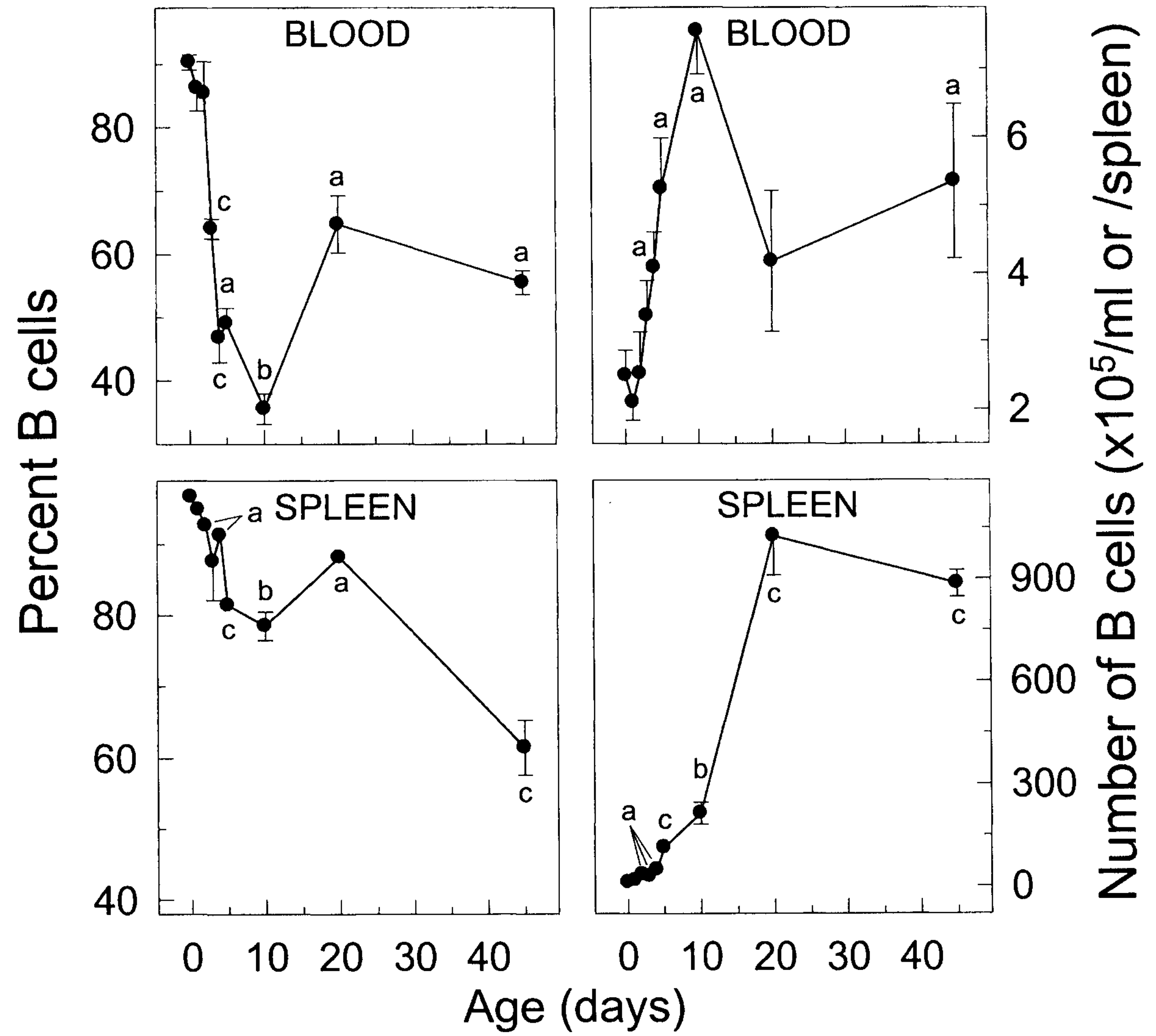

FIGURE 3 Percentage (left panels) and number (right panels) of B cells (CD19) in blood (top panels) and spleen (bottom panels) of CD-1 mice (male and female) at various ages during postnatal development. Data points are mean±SE of 6 samples. Construction of pools is described in Figure 1. Statistical differences ($p < 0.05$) among age groups are indicated by "a" versus d0 and 1, "b" versus days 0–4, 20 and 45 days of age (except percent in blood on d45), "c" is compared to all previous age groups (except absolute numbers in spleen on d20). For this data set df=8, one-way ANOVA with Duncan's multiple range test generated an F statistic >28.7, while Kruskal-Wallis test with Chi Square analysis was >27. At some ages, symbols obscure SE bars memory T cells followed a similar profile as that in blood but represented a greater percentage of the T helper cell population (Figure 5, left bottom panel). By d20, a repertoire of memory T helper cells is well established compared to the nadir on d5. The proportion of naïve T helper cells in spleen first peaked on d3, then gradually rose to d10 and finally declined by d45 (ANOVA, $p<0.05$). Thus as early as d4, naïve T helper cells have achieved adult proportions in both blood and spleen.

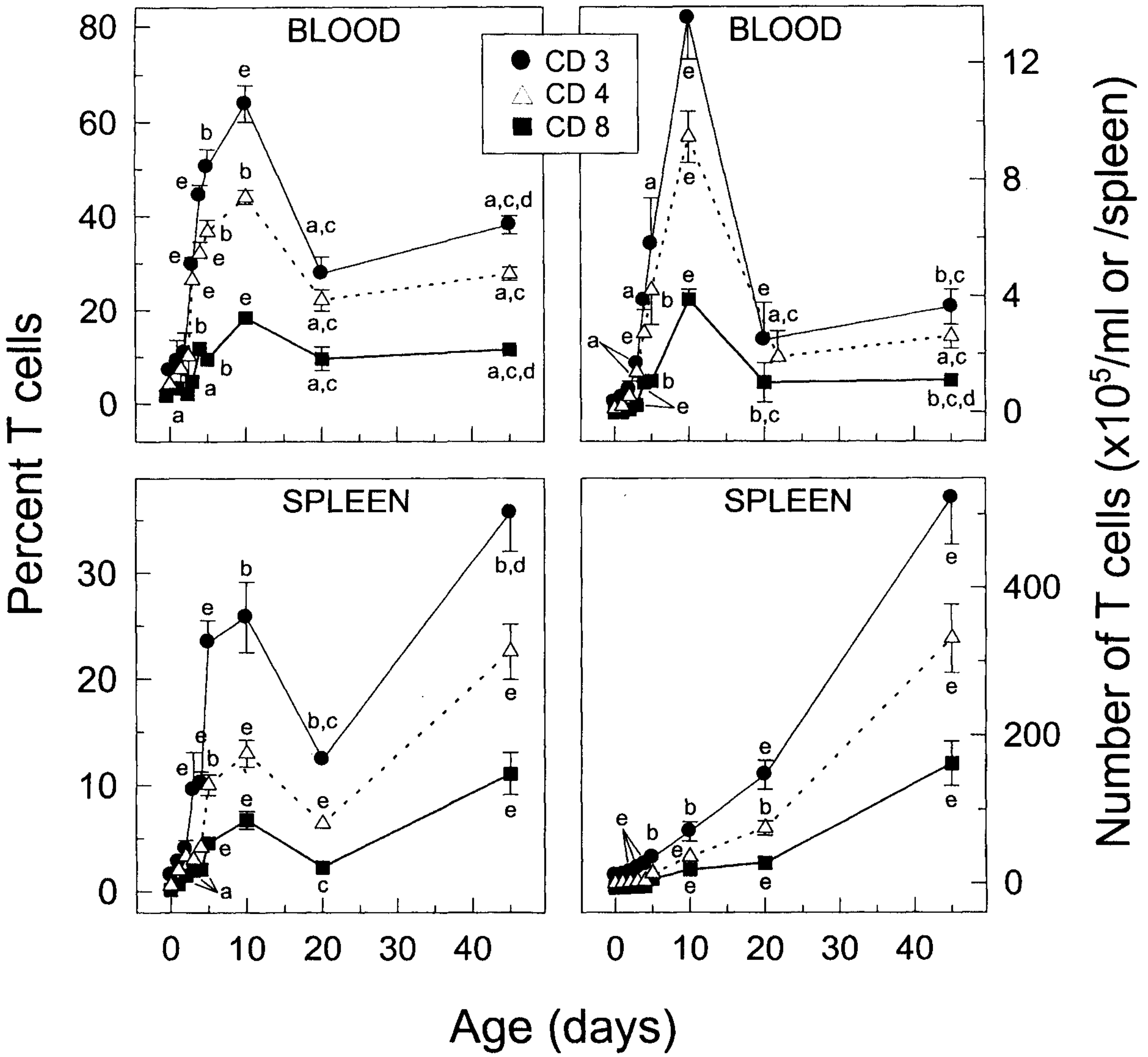

FIGURE 4 Percent (left panels) and numbers (right panels) of lymphocytes (T, CD3; Th, CD3CD4; Tc/s, CD3CD8) in blood (top panels) and spleen (bottom panels) of CD-1 mice (male and female) at various ages during postnatal development. Data points are the mean±SE of 6 samples. Construction of pools is described in Figure 1. Statistical differences ($p < 0.05$) among age groups are indicated by "a" versus d0, "b" versus 0–3 days of age, "c" versus d10, "d" versus d20 and "e" versus all previous ages (except absolute number of CD3 in blood on d20 versus d3). CD4 and CD8 values on days 1–5 are significantly different from previous age groups. For this data set (df=8), one-way ANOVA with Duncan's multiple range test generated an F statistic >27.4, while Kruskal-Wallis test with Chi Square analysis was >40.1. At some ages, symbols obscure SE bars

Attainment of a pool of naïve and memory T helper cells is a critical milestone of immune function maturation, ensuring a large diversity of available repertoires. These two cell types demonstrated similar developmental profiles (Figure 5, right panels). In blood, naïve T helper cells increased 32-fold between d3 and d10 (ANOVA, $p<0.05$); then declined 2-fold by d45. The number of memory T helper cells followed the same pattern. Still, numbers of naïve and memory T helper cells are relatively low in circula-

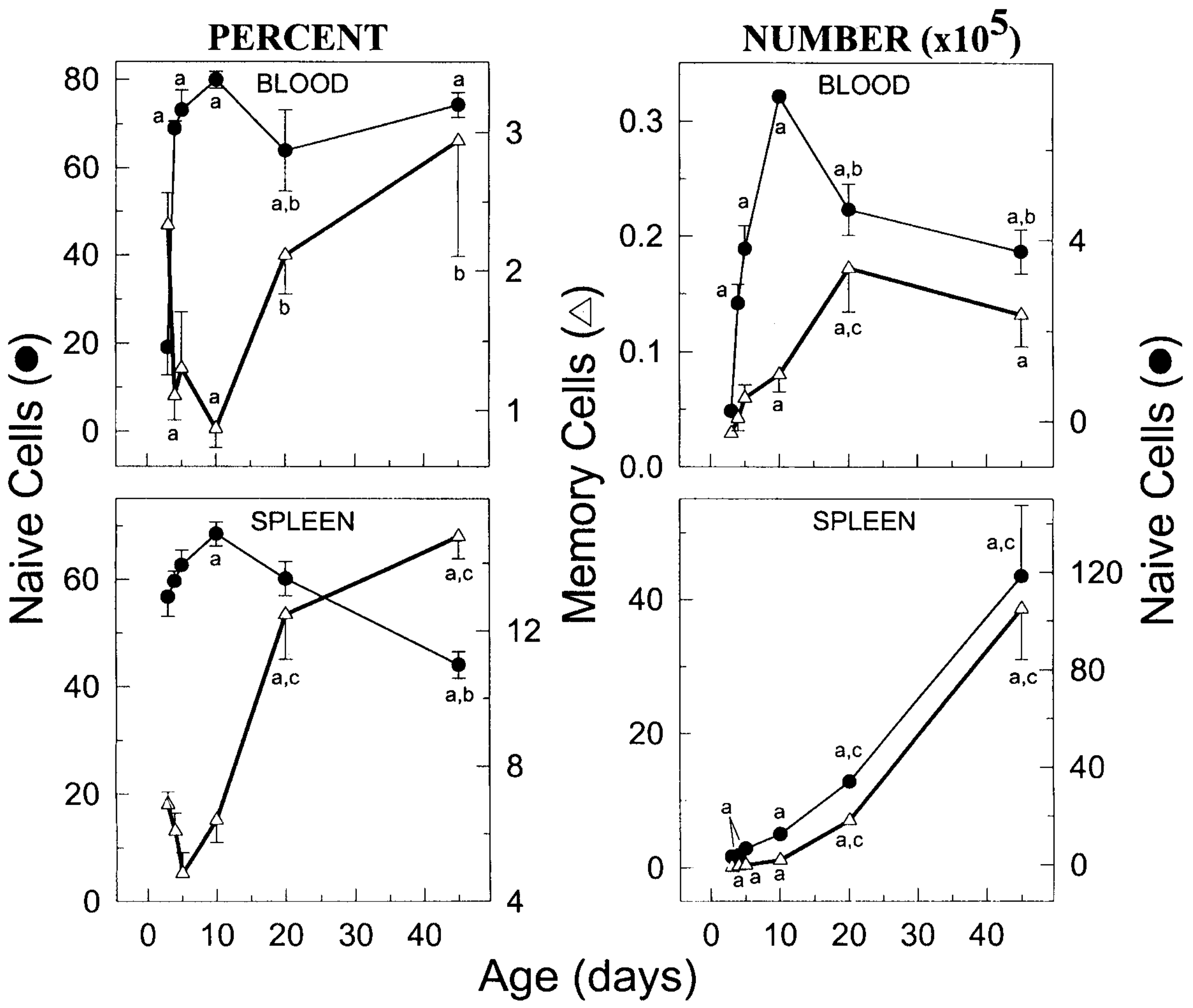

FIGURE 5 Percent (left panels) and numbers (right panels) of naïve (closed circles; $CD4^{pos}CD62^{pos}CD44^{low}$) and memory (open triangles; $CD4^{pos}CD62^{neg}CD44^{high}$) T helper cells in blood (top panels) and spleen (bottom panels) in CD-1 mice (male and female) during postnatal development. Data points are mean±SE of 6 samples. Construction of pools is described in Figure 3.1; note d0–2 were not evaluated. Details of flow cytometric acquisition and analysis are described in Materials and Methods. Statistical differences ($p<0.05$, df=5) among age groups are indicated by "a" versus d3, "b" versus 10. "c" indicates $p<0.05$ compared to all previous age groups. For this data set (df=8), the one-way ANOVA with Duncan's multiple range test generated an F statistic >4.6, Kruskal-Wallis test with Chi Square analysis was >23.3. At some ages, symbols obscure SE bars tion compared to that in spleen (Figure 5, right bottom panel). Importantly, numbers of naïve and memory T helper cells increased exponentially with age (ANOVA, $p<0.05$). Thus, the spleen, a secondary lymphoid organ, appears to be an important residence for both naïve and memory T helper cells.

### Comparison of Immunophenotype Maturation in C57BL/6 and CD-1 Mice

A similar developmental profile was found in C57BL/6 mice for all immune cell phenotypes compared to that in CD-1 mice. However, leukocytes and

TABLE I Age-related changes in B (CD19), NK (NK1.1), T (CD3), Th (CD3CD4), and Tc/s (CD3CD8) cells in blood and spleen of C57Bl/6 mice. Data are expressed as mean percent (±SE) of cells in blood and spleen. $p<0.05$ indicated by letter versus d0 (a), d2 (b), d3 (c), d5 (d), d10 (e), or d20 (f). Note the nadir in blood B cells and peaks in blood T cells on d5, in advance of those in CD1 mice (shaded)

| | **CD19 % Mean (SE)** | | **NK % Mean (SE)** | | **CD3 % Mean (SE)** | | **CD4 % Mean (SE)** | | **CD8 % Mean (SE)** | |
|---|---|---|---|---|---|---|---|---|---|---|
| **Age (days)** | **Blood** | **Spleen** | **Blood** | **Spleen** | **Blood** | **Spleen** | **Blood** | **Spleen** | **Blood** | **Spleen** |
| **0 n=5** | 57.2 (2.7) | 59.0 (4.1) | NT | NT | 9.5 (1.5) | 11.4 (2.3) | 4.7 (1.1) | 2.9 (1.3) | 3.7 (0.9) | 3.6 (0.9) |
| **2 n=4** | 63.6 (3.3) | 65.3 (3.2) | 6.6 (0.8) | 3.8 (1.1) | 18.3 (4.3) | 13.4 (2.3) | 16.1 (5.8) a | 4.9 (1.7) | 4.6 (0.7) | 2.1 (1.9) |
| **3 n=3** | 49.6 (7.2) | 69.3 (8.4) | 4.3 (0.6) | NT | 25.8 (5.3) a | 14.0 (5.5) | 18.5 (3.2) a | 7.0 (2.1) | 6.5 (0.7) | 3.7 (2.1) |
| **4/5 n=9** | **22.7 (1.5) abc** | 52.3 (17.1) | 3.0 (0.3) b | 4.5 (0.5) | **40.2 (3.6) abc** | 9.6 (1.1) | **39.7 (1.7) abc** | 4.4 (1.3) | **11.2 (0.9) abc** | 1.2 (0.2) |
| **10 n=5** | 49.0 (6.8) d | 65.7 (6.4) | 2.74 (0.5) b | 2.6 (0.5) | 31.3 (1.9) ab | 18.7 (2.9) | 19.8 (1.4) ad | 10.0 (1.0) ad | 8.3 (0.7) abd | 4.6 (0.6) |
| **20 n=3** | 37.4 (7.6) b | 54.4 (15.3) | 2.8 (0.6) b | 2.5 (0.2) | 19.8 (2.1) e | 21.6 (5.8) d | 12.4 (0.8) d | 12.4 (3.3) bcd | 7.4 (0.5) ad | 9.3 (6.6) abcde |
| **45 n=3** | 58.9 (6.6) bdf | 53.8 (6.2) | 3.7 (0.2) b | 2.6 (0.1) | 26.3 (2.3) ad | 31.1 (2.4) abcde | 14.7 (1.3) ad | 17.7 (1.1) bcdf | 9.1 (0.5) ab | 9.9 (0.0) abcde |

lymphocytes of these inbred mice developed somewhat later; in blood the peak was detected at d20 (the next time point investigated) in contrast to d10 observed in the CD-1 strain. In spleen, the profiles for leukocytes and lymphocytes were identical for both strains; however, absolute numbers in the inbred mice were about half of those in CD-1 mice (data not shown). In blood, the percentage of NK, B, T and T cell subsets also had similar fluctuations as those observed in the random-bred mice (Table I). The major exception was that, in blood, the nadir for B cells and peak for all T cell subset percentages occurred by d5 rather than d10. In blood the number of B, T, and T cell subsets showed two peaks, a small peak at d5 and a greater peak at d20 (data not shown). In the spleen, the C57BL/6 mice had T cell profiles similar to CD-1 mice, but the age-related differences in B and NK cells were not observed.

### DISCUSSION

The findings indicate that traffic by immune cells into circulation, from primary to secondary lymphoid organs like the spleen, is a developmental milestone in the mouse. The marked increase in several T and B cell populations occurring in the first 10 days after birth are likely a reflection of a heightened production by and subsequent exportation of new cells from the primary lymphoid organs, thymus and bone marrow, respectively. This early activity results in populating secondary lymphoid compartments, creating depots of competent cells as potential recruits for future immune reactions. The majority of this highly active process has occurred by weaning age, a time coincidental with declining maternal serum antibody (transplacental) and withdrawal from milk-borne antibodies and cells.

Early exportation of T and B cells into circulation produces an exponential rise in cell counts by postnatal d10, and thereafter, concentrations of most cell populations change gradually until they approximate adult levels by d20. Significant numbers of NK, B, total T, and T cell subsets are not found until d20, and importantly memory T helper cells are at critically low levels. This profile partially defines the neonatal window for immune system immaturity in the mouse, a period during which the infant depends on passively acquired maternal immune components for protection. The appropriation of sufficient numbers of mature immunophenotype proportions and numbers by 20 days establishes precursors essential to providing independent, mature immune function to the offspring. Attaining this critical threshold of immunophenotypes likely signals the closing of the "window" of compromised inflammatory reactivity and eliminates the bias towards immunotolerance in neonates. Over 40 years ago, Billingham, Brent and Medawar (1953) first described this window of immaturity in allogeneic acceptance by the neonatal mouse. A more recent investigation by Alferink *et al.* (1998) has found that neonatal induction of tolerance requires migration of naïve T cells to peripheral tissues. The present data are consistent with this time-table; increased trafficking and the prevalence of naïve cells (no memory cells) in blood and spleen for the first 10 days apparently underlies the recognized predisposition to allograft tolerance. Only after the occupation of lymphoid compartments by naïve T helper cells is begun does memory cell development begin.

Development of critical numbers of memory T helper cells by day 20, especially in the spleen, may result from pathogenic challenge occurring during the neonatal period. Of interest is the observation that a significant developmental rise in T helper cells occurs in germ free mice (Forni *et al.*, 1988). This may indicate that much of the force driving development of the immune system is derived from exposure to alloantigens encountered *in utero* (Claas *et al.*, 1988; Piotrowski and Croy, 1996) rather than postnatal exposure to pathogens. Unfortunately, in the study of Forni *et al.* (1988), T helper cell differentiation was not assessed, and therefore it is not known whether memory T helper cell numbers increase in absence of xenogeneic challenge. In the present study, memory T helper cells differentiate before d20 (accounting for 12% of all T helper cells; Figure 5). Postnatal memory T helper development may be the result of exposure to both somatic self and xenogeneic epitopes, a consequence of peripheral education of thymic emigrants (De Albuquerque *et al.*, 1994; Thanchot & Rocha, 1997; Alferink *et al.*, 1998). Therefore, differentiation of immunologic memory may be considered part of a mechanism terminating neonatal acceptance of antigens as "self."

Comparisons between random-bred CD-1 and inbred C57BL/6 strains indicate that differences in immunophenotype development are small and may not account for the enhanced inflammatory immune cell activity by C57BL/6 mice (Krishnan *et al.*, 1996a). Developmental profiles for the proportion of most lymphocyte phenotypes were similar between strains. However, absolute numbers of lymphocytes and WBC in the spleen of C57BL/6 mice are about half that of CD-1 mice at each time point studied. In contrast, blood cell concentrations at the neonatal peak were equivalent to the CD-1 strain. C57BL/6 leukocyte and lymphocyte numbers in blood, beyond the neonatal rise on day 20, achieved levels 3.7-fold higher and equivalent, respectively, to those observed at the same postnatal day in CD-1 mice. In addition, exodus by T cells from the thymus appears to peak earlier in C57BL/6 than CD-1 mice. The rate of rise in circulating and splenic lymphocytes was initially the same between strains but then abbreviated in the C57BL/6 inbred animals. A temporal advance in attaining an adult complement of immunophenotypes in blood and spleen in C57BL/6 mice is consistent with premature closure of the window of immaturity that brings about full immune response early in the development of this immunologically aggressive strain. Comparison of this mouse strain with random-bred CD-1 mice indicates that early thymic exportation of T helper cells underlies advanced adult-like immune competence. In contrast, delayed thymic exportation to secondary lymphoid organs prevents competent immune reactivity (Yagi *et al.*,

1996) and is predicted to forestall closure of the window of immune system immaturity. Thus, appropriation of sufficient numbers of mature immuno-phenotypes by d20 may be conceived to establish precursors essential to mature immune function.

The observation that a full complement of lymphocytes is established by weaning seemingly contrasts with previous reports. King *et al.* (1991) have reported that splenic B and T cell subsets only gradually attain adult proportions over the first 35 postnatal days, implying that the young host might be sub-optimally protected between weaning (d20) and d35. At first glance, their report appears to differ from ours (we found a sharp increase in T cell proportions within the first ten days). While a direct comparison with their study was not possible (they analyzed cells relative to total leukocytes rather than lymphocytes), analysis of our data with their methodology revealed that only a gradual rise in T helper cells from birth to adulthood could be demonstrated. Thus, profiles of the two studies are congruent. These data, measured as proportions of total lymphocytes and absolute numbers, indicate and support other reports (Gabor *et al.*, 1997; Modigliani *et al.*, 1994), that thymic exportation of T cells exponentially increases between d3 and d10. The increase in spleen cellularity during the neonatal period, therefore, is primarily due to thymic exportation rather than *in situ* proliferation (Modigliani *et al.*, 1994). Evidence collectively supports the hypothesis that an increase in rate and amplitude of thymic exportation during the first 2 weeks is a critical step in establishing a mature cellular immune system.

Lymphocytic population of splenic compartments appears to continue beyond the neonatal period. Using transgenic cells, Thanchot and Rocha (1997) traced a continuous exportation of naïve T helper cells into splenic compartments well into adulthood. Yet emigration in the adult was maintained at substantially lower levels than in neonates. Continued exportation of T cells to the periphery beyond d20 is required for maintenance of immunological memory (De Albuquerque *et al.*, 1994). Profiles of T helper subsets in the current study are consistent with these observations; naïve T cell proportions in blood and spleen are antecedent to increases in memory cells (Figure 5; left panels). Therefore our data confirm and further define the dynamics of lymphocyte trafficking from primary to secondary lymphoid organs, particularly in the critical first few days postpartum.

To understand the significance of neonatal cell trafficking and the potential impact of establishing specific immune cell populations on the ability of a host to defend itself, proportions and numbers of each population were considered together. T cell profiles in spleen reveal a bimodal distribution. The first peak, occurring before d20, is a result of thymic output without peripheral expansion (cell trafficking, seen in the blood profile) and is a pool of peripheral T cells representing a diversity of available repertoires (Modigliani *et al.*, 1994). However, the second peak, noted after d20, indicates that continued immigration is accompanied by significant clonal expansion (De Albuquerque *et al.*, 1994). A rapid gain in splenic memory T helper cells (Figure 5, bottom left panel) establishes a memory repertoire, permitting delivery of the robust immune responses of an immunocompetent adult while maintaining the original repertoire. Proportionally, NK cells in both blood and spleen demonstrate changes within a narrow range (3%). However, absolute numbers in the adult are 5-fold and 280-fold greater than newborn numbers in blood and spleen, respectively. Clearly, a better picture of lymphocyte population dynamics is obtained when both of these parameters are assessed concurrently.

During the first postnatal days, memory T helper cells are sparse and unlikely to amplify inflammatory responses *in vivo*. Large proportions of B and naïve T helper cells predispose conditions for alloantigen acceptance (Beck *et al.*, 1994). This pattern of peripheralization, however, is subject to genetic influence, a phenomenon observed in C57BL/6 mice. Advanced peripheralization of phenotypes, while not explaining the inflammatory bias of this strain, suggests premature closure of immaturity compared to CD-1 mice. Based on observed differences in immune capabilities of clinical cases and two mouse models, selective advancement (or delay) of this normal process of neonatal immune development in predictable and quantitative ways could be advantageous. That is, an infant's vaccination schedule or immunosuppressive therapy for organ transplantation could be optimized based on the relative state of immune competence and preponderance of T helper cell functional capacity to develop tolerance or aggression.

Immune system performance is the summation of an inventory of immune and accessory cells and their functional capacities. This study has examined a part of that picture, definition of lymphocyte populations occupying blood and spleen compartments between birth and adulthood. Assessment of the functional capacity of these cells during infancy is a logical next step, and is the focus of our current investigations.

### MATERIALS AND METHODS

### Animals

Timed-pregnant CD-1 mice, a random-bred strain, and timed pregnant C57BL/6 mice, an inbred strain, were purchased from Charles River Laboratories (Wilmington, MA) and Jackson Laboratories, Bar Harbor, ME) respectively. The evaluation was performed on the progeny. All mice were housed individually in small plastic cages with microfilters and wood bedding. The mice in cages were maintained in microflo ventilated racks (Allentown Caging Equipment, NJ) in the same room on a 12/12-hour light/dark cycle. The pregnant animals were fed high protein diet, Harlan Teklad 7004/S-2335, (Harlan Teklad, Wisconsin). Compared to the diversity of immune responses expected from the CD-1 mice, adult C57B1/6 mice predominantly express T helper 1 immune responses as evidenced by rapid rejection of male skin isografts by females (Gasser and Silvers, 1971), strong reaction to PHA-stimulation (Hellman and Fowler, 1972) and resistance to both anaphylactic shock (Treadwell, 1969) and *Leishmania major* infection (Krishnan *et al.*, 1996a; Krishnan *et al.*, 1996b).

From birth (d0) to d 5, and again on d10, mice were decapitated and blood collected from the body into heparinized capillary tubes as pools consisting of randomly-sorted pups from different litters. Each group of mice from ages 0 to 10 days consisted of a pool of 5–14 pups/pool and each age group consisted of 6 pools. Post-weaning (days 20 and 45), blood was obtained from individuals by cardiac puncture after pentobarbital anesthesia (40 mg/kg body weight). Spleens were collected from blood donor animals and were grouped as described above. Each spleen was excised, devoid of mesenteric fatty tissue, weighed, and placed in RPMI 1640 medium, at room temperature, until lymphocyte harvest (<30 min). Lymphocytes were collected by gently pressing the tissue through a fine nylon mesh sieve. Leukocytes in blood and spleen cell suspensions (pools of spleen from early ages) were counted (Unopette, Becton Dickinson, Franklin Lakes, NJ). Each group of mice from day 20 consisted of 6 pools of blood and spleens of two spleens/pool, and for the 45-day old mice, each group consisted of 6 individuals. Adult values were assigned to 45-day old mice. At this age, the animals are postpubertal and reproductively mature. Functionally, as reviewed by Mosier and Cohen (1975), T cell proliferative activity in response to mitogens (PHA and ConA) and alloantigens does not mature before the third week after birth. Proliferative responses by splenic T and B cells are also not fully developed until 3–4 weeks of age. Capacity for T cell-dependent antibody production is delayed to 6 weeks of age while cytokine production is not mature until d45 (Adkins *et al.*, 1993).

### Immunophenotyping

Lymphocyte preparations were stained with fluorochrome-labeled monoclonal antibodies for acquisition and analysis by flow cytometry using standard laboratory practice (Raes *et al.*, 1993; Beck *et al.*, 1994; Patrick *et al.*, 1984; and Bray and Landy, 1989). Briefly, cell preparations from whole blood and spleen (0.1 ml, $1 \times 10^5$ cells/tube) were mixed with 10 μl labeled monoclonal antibodies (mAb). A monoclonal antibody panel was constructed for two-(fluoroisothiocyanate (FITC)/phycoerythrin) and three-color immunophenotyping (FITC/Phycoerythrin/Cy-Chrome): for two color, CD19/CD3,

CD4/CD3, CD8/CD3 and CD3/NK1.1 (it was determined that both C57/BL/6 and CD-1 strains express this allele), and for three color, CD62L/CD44/CD4. All monoclonal antibodies were obtained from PharMingen (San Diego, CA), except CD3*FITC obtained from Caltag Laboratories (San Francisco, CA). After incubation (15 min, 4°C), erythrocytes were lysed and the samples centrifuged at 300 × g for 5 min. The supernatant was aspirated and the cell pellet resuspended in 300 μl PBS.

Cells (5000) were acquired and analyzed on a Fluorescence-Activated Cell Sorter with Consort 32 software (FACSort, Becton Dickinson, San Jose, CA). Using side (SSC) and forward (FSC) light scatter parameters, only two morphologically distinct cell populations could be observed in the blood and spleen of newborn mice, lymphocytes and granulocytes. In older pups, a sparse monocyte population was discerned by day 5. Because lymphocyte and monocyte populations in the youngest animals overlap extensively on morphological analysis, T and B cells (CD3 and CD19) were backgated to allow definition of the lymphocyte population.

Through this gate, 5000 events were acquired for CD3, CD3CD4, CD3CD8, NK and 9 cells. Analytical controls included unstained cells (autofluorescence) and cells with isotype antibody (R-PE rat IgG1, κ isotype standard; R-PE hamster IgG isotype standard, group 1 λ; FITC rat IgG2b, κ isotype standard; FITC rat IgG2a, κ isotype standard; FITC rat IgM, κ isotype standard (to evaluate nonspecific staining). Furthermore, a purified rat anti-mouse CD16/CD32 (FcgIII/II receptor) was used as a blocking antibody to ensure lower levels of nonspecific binding (from NK, monocytes, macrophages, granulocytes and mast cells). To quantify naïve and memory T helper cells (Barrat *et al.*, 1995; Sprent and Tough, 1994; Mocci and Coffman, 1997) co-expressing CD4, CD62L and CD44 (3-color), acquisition and analysis were performed on gated CD4 cells (FL2/SSC plot) through a morphological gate excluding monocytes and debris (SSC/FSC plot). Events through this dual gating scheme were analyzed for the expression of CD62L and CD44 on a FL1/FL3 fluorescent dot plot.

The proportions of lymphocyte populations (CD3, CD3CD4, CD3CD8, NK, CD19) were expressed as percentages normalized to the lymphocyte purity (CD19+CD4+CD8+NK) within the analysis gate. Average lymphocyte purity within the morphological gate in the SSC/FSC plot were 61.3, 60.1, 80.7, 85.1, 73.3, 85.0, 90.1, 83.4, 91.7% for blood and 78.5, 79.1, 90.1, 84.7, 78.3, 63.8, 76.0, 76.1, 86.3% for splenocyte samples at ages 0, 1, 2, 3, 4, 5, 10, 20, and 45 days, respectively. This normalization allowed for the exclusion of debris, other non-lymphoid cells (mainly monocytes) and γδ T cells found in the analysis gate. The consistency of results using this normalization was empirically tested by decreasing lymphocyte purity in whole blood analysis of samples from other age groups not requiring normalization; this test revealed a coefficient of assay variation of 1.4%. Normalization was not required for proportions of T helper cells analyzed for CD62L and CD44 expression.

Absolute numbers of each population were generated from white blood cell counts (WBC). Unmodified lymphocyte percentage, i.e., sum of percentages of CD19, CD3 and NK cells relative to total leukocytes and the proportion of CD3, CD3CD4, CD3CD8, NK1.1, CD19 subsets, relative to all lymphocytes. Absolute numbers of T helper cells analyzed for expression of CD62L and CD44 were generated using the leukocyte counts and the percentage of CD3CD4 cells relative to all leukocytes. Cell numbers in spleens were expressed as cells per whole organ (not per gram tissue weight) so as to illustrate the significant increase in total cells accumulating in this secondary lymphoid organ.

***Acknowledgements***

The authors thank Charles Ducsay, PhD and Daila Gridley, PhD for critical review of this work, as well as Cathy Hisey, Catherine Gaffney, Judy Holbeck, and Long Tran for technical assistance, and Grennith Zimmerman, PhD for guidance in the statistical analyses. This work was supported in part by a seed grant from Loma Linda University and in part by an intramural grant from Loma Linda University Medical Center.

### *References*

Adkins B., Ghanei A. and Hamilton K. (1993). Developmental regulation of IL-4, IL-2 and IFN-γ production by murine peripheral T lymphocytes. Journal of Immunology **151**:6617–6626.

Alferink J., Tafuri A., Vestweber D., Hallmann R., Hammerling G.J., and Arnold B. (1998). Control of neonatal tolerance to tissue antigens by peripheral T cell trafficking. Science **282**:1338–1341.

Barclay A.N., Brown M.H., Alex Law S.K., McKnight A.J., Tomlinson M.G., and van der Merwe P.A. (1997). The architecture and interactions of leukocyte surface molecules. In The Leukocyte Antigen FactsBook, Ed.(San Diego:Academic Press), p. 101–129.

Barrat F., Haegel H., Louise A., Vincent-Naulleau S., Boulouis H.J., Neway T., Ceredig R., and Pilet C.. (1995). Quantitative and qualitative changes in CD44 and mel-14 expression by T cell in C57Bl/6 mice during aging. Research Immunology **146**:23–34.

Beck R., Lam P.O. and Tang P.R. (1994). Comparison of cord blood and adult blood lymphocyte normal ranges: a possible explanation for decreased severity of graft versus host disease after cord blood transplantation. Immunology and Cellular Biology **72**:440–444.

Bikoue A, D'Ercole C., George F., Dameche L., Mutin M., and Sampol J. (1997). Quantitative analysis of leukocyte membrane antigen expression on human fetal and cord blood: normal values and changes during development. Clinical Immunology Immunopathology **84**:56–64.

Billingham R.E., Brent L. and Medawar P.B. (1953). Actively acquired tolerance of foreign cells. Nature **172**:603–606.

Bluestone J.A., Cron R.Q., Barret T.A., Houlden B., Sperling A.I., Dent A., Hedrick S., Rellahan B., and Matis L.A.. (1991). Repertoire development and ligand specificity of murine TCRγδ cells. Immunology Reviews **120**:5–33.

Bray R.A. and Landay A.L. (1989). Identification and functional characterization of mononuclear cells by flow cytometry. Archives of Pathology and Laboratory Medicine **113**:579–590.

Claas F.H., Gijbels Y., van-der-Velden-de-Munck J., and van-Rood JJ. (1988). Induction of B cell unresponsiveness to noninherited maternal HLA antigens during fetal life. *Science*. **241**: 1815–1817.

De Albuquerque D.A., Aroeira L.S., Williams O., and Mengel J. (1994). The development of humoral immunological memory to a T-cell-dependent antigen requires thymic emigrant cells. Reserque Immunologie **1445**:185–195.

Forni L., Heusser C. and Coutinho A. (1988). Natural lymphocyte activation in postnatal development of germ-free and conventional mice. Annals of the Institute Pasteur Immunology **139**:245–256.

Gabor M. J., Scollay R., Godfrey D.I. (1997). Thymic exportation is not influenced by the peripheral T cell pool. European. Journal of Immunology. **27**:2986–2993.

Gasser D.L. and Silvers W.K. (1971). Genetic basis of male skin rejection in mice. Transplantation **12**:412–414.

Han P, Hodge G., Story C., and Xu X. (1995). Phenotypic analysis of functional T-lymphocyte subtypes and natural killer cells in human cord blood: relevance to umbilical cord blood transplantation. British Journal of Hematology **89**:733–740.

Hellman A. and Fowler A.K. (1972). Studies of the blastogenic response of murine lymphocytes. III. Specific viral transformation. Proceedings of the Society of Experimental Biology and Medicine **141**:106–109.

King L.E., Morford L.A., Gibbons L.P., and Fraker P.J. (1991) Flow cytometric analysis of the expression of murine B and T surface markers from birth to adulthood. Immunology Letters **31**:73–78.

Krishnan L, Guilbert L.J., Russell A.S., Wegmann T.G., Mosmann T.R., and Belosevic M. (1996a). Pregnancy impairs resistance of C57Bl/6 mice to Leishmania major infection and causes decreased antigen-specific IFN-gamma response and increased production of T helper 2 cytokines. Journal of Immunology **156**:644–652.

Krishnan L., Guilbert L.J., Wegmann T.G., Belosevic M., and Mosmann T.R. (1996b). T helper 1 response against Leishmania major in pregnant C57Bl/6 mice increases implantation failure and fetal resorptions. Correlation with increased IFN-gamma and TNF and reduced IL-10 production by placental cells. Journal of Immunology **156**:653–662.

Mocci S. and Coffman R. (1997). The mechanism of *in vitro* T helper cell type 1 to T helper cell type 2 switching in highly polarized Leishmania major-specific T cell populations. Journal of Immunology **158**:1559–1564.

Modigliani Y., Coutinho G., Burlen-Defranoux O., Coutinho A., and Bandeira A. 1994. Differential contribution of thymic outputs and peripheral expansion in the development of peripheral T cell pools. European Journal of Immunology **24**:1223–1227.

Mosier D.E. and Cohen P.L. (1975). Ontogeny of mouse T-lymphocyte function. In Development and aging in organ systems. Federation of the American Society of Experimental Biology. Atlantic City, NJ. April, 1974:137–140.

Nahmias A., Ibegu C., Lee F., and Spira T. (1994). The development of the immune system --importance in the ascertainment of immunophenotypic changes in perinatal HIV infection. Clinical Immunology and Immunopathology **71**:2–7.

Patrick C.W., Swartz S.J., Harrison K.A., and Keller R.H. (1984). Collection and preparation of hematopoietic cells for cell marker analysis. Laboratory Medicine **15**:659–665.

Pittard W.B., Schleich D.M., Geddes K.M., and Sorensen R.U. (1989). Newborn lymphocyte subpopulations: the influence of labor. American Journal of Obstetrics and Gynecology **160**:151–154.

Piotrowski P. and Croy B.A. (1996) Maternal cells are widely distributed in the murine fetuses in utero. *Biol. Rep.* **54**:1103–1110.

Raes M, Alliet P., Gillis P, Zimmermann A., and Kortleven J. (1993). Lymphocyte subpopulations in healthy newborn infants: comparison of cord blood values with values five days after birth. Journal of Pediatrics **123**:465–467.

Sprent J, and Tough D. (1994). Lymphocyte life span and memory. Science **265**:1395–1400.

Thanchot C. and Rocha B. (1997). Peripheral selection of T cell repertoires: The role of continuous thymus output. Journal of Experimental Medicine **186**:1099–1106.

Tough D.F. and Sprent J. (1998). Lifespan of γ/δ T cells. Journal of Experimental Medicine **187**:357–365.

Treadwell P.E. (1969). The inheritance of susceptibility to anaphylaxis in inbred mice and their hybrid progenies. Journal of the Reticuloendothelial Society **6**:343–53.

Witherden D.A., Kimpton W.G., Abernethy N.J., and Cahill R.N.P. (1994). Changes in thymic export of γδ and αβ T cells during fetal and postnatal development. European Journal of Immunology **24**:2329–2336.

Yagi H., Matsumoto M., Nakamura M., Makino S., Sizuki R., Harada M., and Itoh T. (1996). Defect of thymocyte emigration in a T cell deficiency strain (CTS) of the mouse. Journal of Immunology **157**:3412–3419.

Yamaguchi N., Shimizu S., Hara A., and Saito T. (1983). The effect of maternal antigenic stimulation upon the active immune responsiveness of their offspring. Journal of Immunology **50**:229–238.

